# Diagnostic utility of speech-based biomarkers in mild cognitive impairment: a systematic review and meta-analysis

**DOI:** 10.1093/ageing/afaf316

**Published:** 2025-10-28

**Authors:** Zahra Jafari, Melissa K Andrew, Kenneth J Rockwood

**Affiliations:** School of Communication Sciences and Disorders, Dalhousie University, Halifax, Nova Scotia, Canada; Geriatric Medicine, Dalhousie University, Halifax, Nova Scotia, Canada; Otolaryngology - Head & Neck Surgery, Dalhousie University, Halifax, Nova Scotia, Canada; Psychology and Neuroscience, Dalhousie University, Halifax, Nova Scotia, Canada; Geriatric Medicine, Dalhousie University, Halifax, Nova Scotia, Canada; Geriatric Medicine, Dalhousie University, Halifax, Nova Scotia, Canada

**Keywords:** mild cognitive impairment, subjective cognitive decline, speech analysis, accuracy, sensitivity, specificity, machine learning, automatic speech recognition, systematic review, older adults

## Abstract

**Background:**

Among various tools developed for mild cognitive impairment (MCI) detection, analysing speech features is a non-invasive and cost-effective approach that shows promise for early detection. This review aimed to systematically synthesise and analyse current evidence on the diagnostic utility of speech-based biomarkers for identifying MCI.

**Methods:**

A systematic review and meta-analysis were conducted following Preferred Reporting Items for Systematic Reviews and Meta-Analyses guidelines. PubMed, Scopus, Ovid Medline and PsycINFO databases were searched up to April 2025 without restrictions on language, article status or year.

**Results:**

Of 4432 identified records, 54 peer-reviewed articles met the inclusion criteria. Fixed-effects meta-analyses showed pooled estimates of 80.0% ‘accuracy’ [95% confidence intervals (CI): 70.0%–89.0%, *P* < .001, *n* = 21], 78.0% ‘area under the curve’ (95% CI: 70.0%–86.0%, *P* < .001, *n* = 21), 80.0% ‘sensitivity’ (95% CI: 71.0%–90.0%, *P* < .001, *n* = 22), and 77.0% ‘specificity’ (95% CI: 65.0%–89.0%, *P* < .001, *n* = 15) in differentiating MCI from cognitively unimpaired (CU) individuals. Egger’s regression tests indicated no publication bias (*P* ≥ .299), and the *I*^2^ statistic revealed no heterogeneity across studies (*I*^2^ = 0.00%, *P* = 1.00). Four studies also included a subjective cognitive decline group, reporting significant differences in certain speech features compared to CU.

**Conclusions:**

Speech analysis demonstrates moderate classification performance, with balanced sensitivity and specificity, in distinguishing MCI from CU, suggesting its potential as an accurate and cost-effective diagnostic tool for MCI detection. Further research is needed to address variations in study methodologies, refine speech analysis protocols and validate findings in diverse populations to enhance generalisability.

## Key points

- This review intersects the fields of ageing, neurodegeneration, digital health and data science.
- Speech analysis has a good diagnostic utility to distinguish mild cognitive impairment from cognitively unimpaired status.
- No publication bias was detected, and heterogeneity across studies was minimal.
- Future research should address methodological variations, refine analysis protocols and validate findings in diverse populations.

## Introduction

One of the main causes of dementia, Alzheimer’s disease (AD), develops gradually over years or even decades [1], frequently starting with a silent presymptomatic phase [2, 3]. AD is characterised by a progressive decline in memory, language, executive function and other cognitive domains, hampering independence and quality of life in later stages [1]. Mild cognitive impairment (MCI) and subjective cognitive decline (SCD) are recognised as early cognitive concerns for being at risk, but are often overlooked in clinical settings. MCI has long been recognised as the prodromal stage of AD, with validated tools available for its detection [4]. SCD, a self-reported early concern about cognitive function despite results within normal limits on standard cognitive assessments, may represent the earliest symptomatic manifestation of AD in some cases [5–7]. Population-based studies indicate reports of SCD in 50%–80% of older adults [8]; however, systematic follow-up and risk stratification are less likely to be implemented. Despite traditional practices for AD diagnosis, which rely on medical history taking and comprehensive examinations, current clinical research promotes a multifaceted approach that combines cognitive assessments, neuropsychological testing and the use of biomarkers [9]. Examples of such multimodal biomarkers include positron emission tomography (PET) and magnetic resonance imaging to assess brain atrophy, electroencephalography (EEG) and functional near-infrared spectroscopy to study altered neural oscillations and brain functional connectivity and cerebrospinal fluid (CSF) analysis to measure amyloid-beta, tau and phosphorylated tau levels [3, 10–12]. Despite the high precision of adopting a multimodal approach for early diagnosis, factors such as cost, invasiveness and geographical constraints can limit access, highlighting the need for clinically accurate, accessible, scalable and cost-effective screening methods to bridge the barriers for early cognitive decline detection.

Speech analysis is a non-invasive tool extensively used as an accessible approach for cognitive decline detection [5, 13, 14]. Speech production is a complex cognitive process involving memory retrieval, semantic processing, syntactic planning and motor execution [15, 16]. This high level of cognitive demand renders subtle changes in speech patterns a promising and practical marker for the early detection of cognitive decline. Researchers have explored a range of speech features as biomarkers, including lexical diversity, syntactic complexity, acoustic properties and semantic content [5, 13, 14, 17–19]. In addition, recent advances in automatic speech recognition (ASR) technologies [5, 13, 14, 17–24], natural language processing (NLP) models [25, 26] and machine learning (ML) algorithms [14, 18–20, 22, 23, 27, 28] have significantly increased the use of speech biomarkers for MCI early detection. For example, studies have employed ML classifiers such as support vector machines (SVMs), random forests and logistic regression to distinguish individuals with MCI from cognitively unimpaired (CU) peers [18, 22, 29, 30].

In addition to benefiting from artificial intelligence (AI)-based technologies, speech biomarkers offer unique advantages, including their non-invasive nature, cost-effectiveness and potential for remote administration, repeated assessments and large-scale cognitive screening [5, 7, 13]. Previous reviews have shown that speech analysis techniques can effectively differentiate individuals with AD from CU counterparts [31, 32]. However, a systematic review (SR) and meta-analysis (MA) specifically examining the diagnostic utility of speech-based biomarkers for classifying MCI or SCD from CU are lacking. This review, situated at the intersection of ageing, neurodegeneration, digital health and data science, aims to fill this gap by systematically synthesising current evidence on the use of speech features to distinguish individuals with MCI or SCD from CU peers. We hypothesise that speech-based biomarkers have diagnostic utility in differentiating MCI or SCD from CU and may contribute to the early detection of cognitive decline.

## Methods

### Systematic review

#### Search strategy

**Figure 1:**
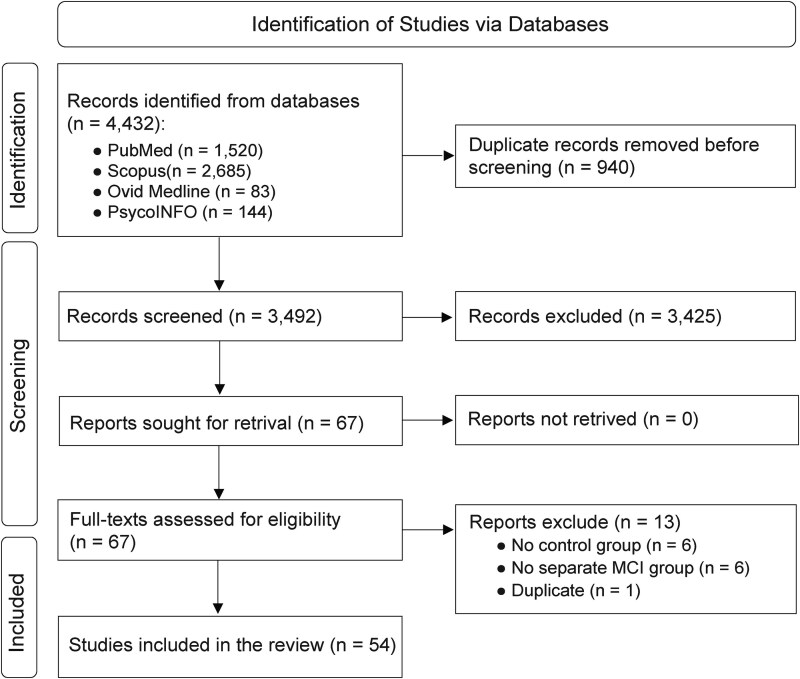
The Preferred Reporting Items for Systematic Review and Meta-Analyses flow diagram details the search and selection process applied during the systematic review. Publications that did not meet participant, intervention, comparator, outcomes, and study design’s criteria were excluded.

We followed the Preferred Reporting Items for Systematic Reviews and Meta-Analyses Statement 2020 (see Supplementary Appendix S1). Four electronic databases were searched up to April 2025: PubMed, Scopus, Ovid Medline and PsycINFO (Figure 1). The search strategy had no restrictions on language, publication status or date. The combined search term ‘mild cognitive impairment’ OR ‘subjective cognitive decline’ OR ‘dementia’ OR ‘Alzheimer’s disease’ AND ‘speech analysis’ OR ‘voice analysis’ was used for the systematic search (see Supplementary Appendix S2). The reference list of included studies was manually checked to identify any missing articles. The SR protocol was established before the review and registered with the PROSPERO International Prospective Register of Systematic Reviews (registration number# CRD420251013299).

#### Inclusion and exclusion criteria

Inclusion and exclusion criteria were defined using the participants, interventions, comparators, outcomes and study designs framework as follows: (P) peer-reviewed articles on individuals with MCI or SCD, (I) collecting speech samples, (C) CU or typical individuals without cognitive impairment, (O) analysing speech features and (S) randomised and observational studies. Reviews, books, case reports, short reports and editorials were excluded.

#### Study selection

Using EndNote software, the search records were evaluated through a two-stage process: title and abstract screening, followed by full-text screening [33].

#### Data extraction

The following data were extracted from the included articles: first author’s name, year of publication, country, study aim, participants’ characteristics, speech tasks, speech features, analysis method and primary findings, including four classification metrics: accuracy, area under the curve (AUC) of the receiver operating characteristic, sensitivity and specificity in distinguishing MCI from CU.

AUC and accuracy are among the most broadly used statistical metrics to evaluate model performance, including in the context of cognitive decline detection. Accuracy refers to the overall proportion of correct predictions. It is the sum of true positives and true negatives relative to the total number of predictions, with 100% accuracy denoting a perfect result and 50% representing performance no better than chance. In contrast, the AUC measures the model’s ability to distinguish between classes, such as MCI versus CU. An AUC closer to 1.0 indicates high discriminatory power, while an AUC of 0.5 may represent random chance. Sensitivity, also known as the true positive rate, reflects the proportion of actual positives correctly identified by the model, with higher values indicating better detection of the target condition. Specificity, or the true negative rate, represents the proportion of actual negatives correctly identified, with higher values indicating accurate recognition of non-target individuals [34, 35].

### Methodological quality of evidence

The Joanna Briggs Institute (JBI) critical appraisal checklist for analytical studies was used to assess the methodological quality of the included articles [33] (see Supplementary Appendix S3). According to the JBI checklist, scores of 0–2, 3–5 and 6–8 indicate weak, moderate and high quality, respectively.

Two reviewers independently assessed the two stages of the systematic search and the studies’ methodological quality, aiming to resolve disagreements by consensus [36].

### Meta-analysis

#### Analysis of effect size

Using RStudio (version 2025.05.1+513), a fixed-effect MA was used to estimate the classification performance of accuracy, AUC, sensitivity and specificity across studies. Standard errors (SEs) for these metrics were calculated using the inverse of the sample sizes in the MCI and CU groups and used to quantify pooled effect sizes. In the MAs, *Z*-scores indicate whether the combined effects significantly differ from zero, and *P*-values represent statistical significance. A forest plot was created to visually summarise individual study estimates and their confidence intervals (CI) [37].

#### Bias assessment and heterogeneity analysis

Both funnel plots and Egger’s test for publication bias were used to examine potential biases in the MAs [38]. The funnel plot is used to identify asymmetry as a sign of publication bias. Egger’s regression test was conducted to formally test this, where a statistically significant *P*-value suggests the likelihood of bias. The *I*^2^ statistic and Tau^2^ values were used to analyse variability among the included studies. The *I*^2^ statistic represents the degree of heterogeneity, with values of 0%, 25%, 50% and 75% representing no, low, moderate and high heterogeneity, respectively. When heterogeneity is substantial (*I*^2^ ≥ 50%), a random-effects model is carried out [39].

## Results

### Literature search

Of the 4432 identified records, 54 peer-reviewed articles were included in the SR. Hand-searching the reference lists of selected articles did not identify any additional relevant studies. The two reviewers were in complete agreement on the final selection of studies.

Table 1 provides a summary of the included studies [5–7, 13, 14, 17–27, 29, 30, 40–75]. Most research samples predominantly came from the USA, followed by Japan, Hungary, Italy, China, Korea and Spain. The selected articles included MCI and CU groups; four also examined an SCD group. Of the 27 studies using ASR, 13 relied solely on ASR, 7 combined ASR with manual transcription and 7 used both ASR and manual transcription. The studies in the last group reported equal or comparable classification results for the two transcription techniques (Table 2). Of the 54 included studies, 35 provided sufficient data for inclusion in MAs of AUC (*n* = 23), accuracy (*n* = 21), sensitivity (*n* = 22) and specificity (*n* = 15) classification metrics. Of the 35 studies included in the MAs, 31 [6, 13, 14, 18–24, 27, 29, 30, 44, 46, 49, 50, 54–56, 58–61, 63, 64, 68, 70, 71, 73, 75] employed ML classifiers and common techniques, such as cross validation (CV) and feature selection (FS), to address overfitting.

**Table 1:** Descriptive characteristics of the studies included in the systematic review.

Descriptive characteristics	Number of studies
Articles included in the systematic review	
MCI	53 [5–7, 13, 14, 17–27, 29, 30, 40–64, 66–75]
SCD	4 [5–7, 65]
Transcription method	
	ASR [13, 18–20, 22–24, 29, 41, 44, 48, 49, 54]
	Both ASR and manual [5, 7, 21, 46, 47, 53, 64]
	ASR followed by manual transcription (combined) [14, 30, 50, 51, 59, 61, 75]
Classification metrics for meta-analyses	
Accuracy	21 [18–23, 27, 29, 41, 49, 53–55, 58, 61, 63, 64, 68, 70, 71, 73]
AUC	23 [13, 14, 20, 22–24, 30, 42, 44, 46–49, 54, 56, 58, 60, 63, 64, 66, 68, 71, 75]
Sensitivity	22 [13, 14, 18, 19, 21–23, 29, 30, 41, 42, 47, 49, 53–55, 58, 61, 63, 64, 68, 71]
Specificity	15 [13, 14, 21–23, 30, 41, 47, 54, 58, 61, 63, 64, 68, 71]
Using remote speech-based testing	4 [5, 23, 29, 56]
Using NLP algorithms	6 [14, 18, 26, 44, 46, 75]
Analysis using ML classifiers	31 [6, 13, 14, 18–24, 27, 29, 30, 44, 46, 49, 50, 54–56, 58–61, 63, 64, 68, 70, 71, 73, 75]
Techniques to address over-fitting	
CV	15 [13, 18, 23, 24, 27, 29, 41, 42, 50, 54, 58, 60, 61, 68, 75]
FS	5 [6, 22, 30, 53, 59]
Both CV and FS	11 [14, 19–21, 46, 49, 55, 56, 70, 71, 73]
Ensemble modelling	3 [27, 44, 56]
Study population	
USA	14 [13, 14, 19, 24, 29, 30, 44, 46, 50, 56, 67–69, 75]
Japan	7 [21, 26, 27, 54, 60, 61, 71]
Hungary	5 [45, 49, 51, 58, 64]
Italy	4 [20, 25, 53, 59]
China	4 [18, 22, 48, 55]
Korea	4 [17, 43, 57, 62]
Spain	3 [6, 41, 52]
Germany	3 [5, 23, 74]
France	2 [70, 73]
UK	1 [7]
Brazil	1 [72]
Ireland	1 [66]
Sweden	1 [63]
Singapore	1 [47]
Belgium	1 [40]
Cyprus	1 [42]
Czech Republic	1 [65]

LR, logistic regression; KNN, k-nearest neighbour; RF, random forest.

**Table 2:** Characteristics of the studies using speech-based analyses to distinguish MCI or SCD from CU.

No	Author year, country	Aim	Participants	Speech tasks	Speech features	Analysis method	Findings
1	Skirrow, 2024 [13] USA	Speech-based testing (ADNI4 in-clinic cohort)	13 with MCI (5f, 75.0y), 44 CU (27f, 73.70y)	ASR transcription Story recall tasks in the Storyteller test battery	Text similarity (G-match), lexical and acoustic (temporal and spectral)	Using G-match to predict MCI by ROC analysis 5-fold CV	Combined AUC = 70.0%, Sen = 85.0%, Spe = 59.0%
2	König *et al.* 2024 [5] Germany	Automated vs. manual transcription	43 with SCD (20f, 72.0y), 14 with MCI (4f, 72.4y), 18 CU (15f, 74.9y)	ASR and manual transcriptions Telephone-based remote immediate and delayed verbal learning, SVF, narrative storytelling tasks	Word count, semantic cluster size, cluster switches, word frequencies	Bayesian Bland–Altman analysis	High levels of agreement between ASR and manual transcriptions Pairwise comparisons were not reported
3	Kleiman, 2024 [14] USA	Using filled and unfilled pause metrics	14 with MCI (9f, 76.64y), 29 CU (21f, 68.28y)	ASR followed by manual transcription (combined) Immediate and narrative recalls, picture description and free response	16 of the best performing features, e.g. total filled pauses, total ‘uh’ pauses, mean latency post ‘uh/um’, word count, etc.	NLP algorithms. ML classifiers: RF, GB, LR, SVM 3-fold 3-repeat CV, FS	AUC = 82.8%, Sen = 43.59%, Spe = 94.59%
4	Kim and Choi, 2024 [17] Koria	Comparing word naming GI	30 with MCI (15f, 71.27y), 41 CU (22f, 72.61y)	Unclear transcription method. SST, naming tasks: K-BNT and K-COWAT	GI and specific word-finding behaviours: substitution, reformulation, repetition, empty words, etc.	Traditional statistics	Significantly lower scores in GI, insertions and word reformulations
5	Huang *et al.* 2024 [18] China	Automated speech analysis for MCI	40 with MCI (23f, 69.90y), 22 CU (10f, 64.95y)	ASR transcription CTPD	Several acoustic (prosodic, spectral, vocal quality) and linguistic (lexical, syntactical, pragmatics)	NLP algorithms. ML classifiers: LR, RF, SVM, GNB, KNN LOSO CV	Accuracy = 80.43%, 76.06% and 77.17% with linguistic, acoustic and combined features Sen = 80.23%, 76.09% and 77.17% with linguistic, acoustic and combined features
6	Chen *et al.* 2024 [40] Belgium	Connected-speech tasks in MCI detection	16 with MCI (12f, 70.3y), 16 CU (12f, 70.0y)	Picture description, story narrative, story recall and 7rocedural narrative tasks	Several lexical-semantic features categorised into RevR, RepR, FPR, WRR, CoreLex, PID, O/CCWR and NLF	Traditional statistics	Only the story recall task differentiated between the two groups
7	Cay *et al.* 2024 [19] USA	Identifying optimal discriminating features	36 with MCI (25f, 67.0y), 23 CU (14f, 69.0y)	ASR transcription. Reading a standard passage (Rainbow)	Several timing, acoustic and similarity (DTW) features	ML classifier: RF CV, FS	Accuracy = 78.3%, Sen = 83.1%
8	Ambrosini *et al.* 2024 [20] Italy	Automated speech analysis for MCI	88 with MCI (33f, 82.8y), 88 CU (39f, 76.5y)	ASR transcription Telling 3 stories about life for 2 minutes, CTPD	Several acoustic features under voice periodicity, shimmer, syllables, pauses and spectral categories	ML classifiers: LR, SVM, CAT 10-fold CV, FS	Accuracy = 80.0%; AUC = 65.0%
9	Yamada *et al.* 2023 [21] Japan	Automatic speech analysis in MCI	46 with MCI (18f, 73.8y), 43 CU (28f, 72.16y)	Both ASR and manual transcriptions Countdown, backward, subtraction, PVF, SVF, CTPD	Acoustic (MFCC), prosodic (pause duration, pitch variation, phoneme rate) and linguistic (total number of information units, number of filter words)	ML classifiers: KNN, RF, SVM, LightGBM LOSO CV, FS	Accuracy = 87.6%, Sen = 88.1%, and Spe = 87.2% High correlation between ASR and manual transcriptions (92%)
10	Wang R *et al.* 2023 [22] China	ASR in MCI detection	41 with MCI (19f, 69.46y) and 41 CU (19f, 69.27y)	ASR transcription Four reading tasks (syllable utterances, tongue twister, diadochokinesis and short sentence reading)	Several acoustic features, e.g. time change rate of F1, interval time between intensity peaks, speech rate, utterance duration, number of silence pauses, etc.	ML classifiers: RF, SVM, NB FS	Accuracy and AUC = 81.0%, Sen = 85.0%, Spe = 83.0%
11	Sch¨afer *et al.* 2023 [23] Germany	MCI detection with linguistic metrics	48 with MCI (NR, 75y), 356 CU (NR, 75y)	ASR transcription Remote speech recordings from RAVLT and SVF	Semantic and temporal aspects	ML classifiers: RF, SVM, extra trees LOSO CV	Accuracy =71.0%, AUC = 73.0%, Sen = 79.0%, Spe = 72.0%
12	Mefford *et al.* 2023 [24] USA	Automated speech analysis for MCI subtypes	18 with aMCI (6f, 70.2y), 15 with MCI (6f, 71.5y), 62 CU (35f, 70.3y)	ASR transcription CTPD	Multiple features: 44 acoustic and 47 linguistic	ML classifiers 5-fold CV	AUC = 0.74 (0.88 and 0.61 for aMCI and non-MCI, respectively.)
13	Martínez-Nicolás *et al.* 2023 [41] Spain	Multi- vs. single-task speech analysis	24 with MCI (NR, 84.12y), 24 CU (NR, 82.5y)	ASR transcription A reading task (novel Don Quixote) and orally repeating a sentence	Several acoustic, rhythm and voice quality features	Stepwise, linear discriminant analysis LOSO CV	Accuracy = 83.3%, Sen = 83.0%, Spe = 93.0%
14	Loizou and Pantzaris 2023 [42] Cyprus	Automated speech analysis for MCI detection	65 with MCI (26f, 82.66y), 95 CU (60f, 74.32y)	A 2-minute interview	55 different acoustic and linguistic features	Traditional statistics	AUC = 91.0%; Sen = 89.0%
15	Kim *et al.* 2023 [43] Korea	Discourse performance in MCI	38 with MCI (16f, 74.11y), 117 CU (45f, 74.88y)	Discourse task, part of the BCCD	Discourse subscales (coherence, cohesion, proposition, pause) and total scores	Traditional statistics	Significantly lower scores in cohesion, pause and total scores.
16	He *et al.* 2023 [6] Spain	Automated classification of cognitive decline	31 with SCD (8f, 69.0y), 39 with MCI (15f, 76.0y), 18 CU (11f, 66.0y)	Describing an imaginary scene	Acoustic (spectral, cepstral, voice quality), prosodic and textual (morpholexical, semantic, syntactic)	ML classifier: RF FS	High diagnostic classification accuracies among various degrees of cognitive decline Cognitively The difference between MCI and CU was not reported
17	Hajjar *et al.* 2023 [44] USA	Developing digital voice biomarkers	114 with MCI (65f, 64.9y), 92 CU (60f, 63.5y)	ASR transcription VF and confrontation naming tasks, CSF Brain imaging	Multiple lexical, semantic and acoustic features	NLP algorithms ML classifiers: LR, NN FS and ensemble models	An AUC of 80.0%, 77.0% and 66.0% for the lexical, acoustic and the Boston naming test, respectively
18 Task	Balogh *et al.* 2023 [45] Hungary	VF analysis in MCI	25 with MCI (7f, 71.72y) and 25 CU (8f, 67.32y)	Phonemic (K, t, a) and semantic (animals, food items, actions) PVF, FVF tasks	Several verbal fluency features, e.g. number and average duration of silent pause, hesitation, irrelevant utterance, etc.	Traditional statistics	AUC, Sen and Spe for silent pauses = 74.0, 76.0, 50.0%; the average length of silent pauses = 70.2, 72.0, 50.0% and the average word transition time = 78.8, 96.0, 62.50%
19	Amini *et al.* 2023 [46] USA	Automated MCI detection (Framingham Heart Study)	387 with MCI (220f, 81.6y), 410 CU (204f, 77.2y)	Both ASR and manual transcriptions Digital voice recording of subjects’ neuropsychological tasks	Semantic	NLP algorithms ML classifiers: LR, MLP 10-fold CV, FS	AUC = 74.4% Comparable results with both ASR and manual transcriptions
20	Zhao *et al.* 2022 [47] Singapore	Voice digital cognitive screener	43 with MCI (31f, 71.3y), 50 (37f, 70.4y) CU individuals	Both ASR and manual transcriptions A semi-automatic conversational robot that asks questions and provides cues	Several verbal recalls and semantic fluency features	Traditional statistics	AUC = 77.0%, Sen = 69.0%, Spe = 70.0% Comparable results with both ASR and manual transcriptions
21	Wang H-L *et al.* 2022 [48] China	Silence duration for MCI detection	95 with MCI (54f, 73.0y), 113 CU (71f, 67.6y)	ASR transcription CTPD	Acoustic: ratio of total silent pause duration to total speech duration	Traditional statistics	AUC = 74.0% Increased silent duration in MCI
22	Vincze *et al.* 2022 [49] Hungary	Automatic MCI detection	25 with MCI (NR, 72.4y), 24 CU (NR, 70.72y)	ASR transcription SSTs: describing two short videos and yesterday’s activities	Several linguistic features, e.g. number of sentences and words, number and frequency of tokens and distinct lemmas, etc.	ML classifier: SVM 5-fold CV, FS	Accuracy = 77.3%, AUC = 84.5%, Sen = 84.0%
23	Tröger*et al.* 2022 [7] UK	Speech biomarkers for MCI	69 with SCD (24f, 62.20), 52 with MCI (19f, 70.29y), 25 CU (15f, NR)	Both ASR and manual transcriptions Speech recorded during RAVLT and VFT	50 speech features	Traditional statistics	The two groups differed significantly in terms of speech features. Pairwise comparisons were not reported Comparable results with both ASR and manual transcriptions
24	Sanborn *et al.* 2022 [50] USA	Automated MCI detection	26 with MCI (17f, 68.58y), 62 CU (42f, 67.81y)	Combined ASR and manual transcription SST, picture description	16 lexical-semantic, e.g. total words, filler words, lexical frequency, speech/filler rate, etc.	ML classifier: LR 5-fold CV	A significant difference in most linguistic features
25	Nishikawa *et al.* 2022 [27] Japan	Automated MCI detection	13 with MCI (NR, 74.3y), 37 CU (NR, 75.2y)	Recorded speech	Acoustic features: MFCC, jitter, shimmer	ML classifiers: SVM, RF, LightGBM 3-fold CV	Accuracy = 87.7% with SCM fine-tuned with ensemble models
26	Liang *et al.* 2022 [29] USA	Remote VASs for MCI detection	18 with MCI (9f), 22 CU (7f) ≥ 65y	ASR transcription Performing 30 commands of the remote Alex device	163 markers from voice commands of a VAS, Amazon Alex	ML classifiers: RF, SVM, NNs, decision trees LOSO CV	Accuracy = 63%; Sen = 73.0%
27 feature	Kálmán *et al.* 2022 [51] Hungary	Temporal markers for MCI	14 with MCI (8f, 72.36y), 19 CU (14f, 74.47y)	Combined ASR and manual transcription SSTs	15 acoustic features, e.g. utterance length, articulation tempo, speech tempo, etc.	Traditional statistics	AUC, Sen and Spe were reported for each acoustic feature
28	Chapin *et al.* 2022 [52] Spain	Linguistic profile of MCI	35 with MCI (12f, 68.51y), 35 CU (23f, 68.11y)	CTPD	Three categories of linguistic features: nominals, verb phrases and clausal domains	Traditional statistics	Fewer adjunct clauses and adverbial adjuncts.
29	Bertini *et al.* 2021 [53] Italy	Automatic speech analysis in MCI	32 with MCI (16f, 64.32y), 48 CU (24f, 61.60y)	Both ASR and manual transcriptions Response to three questions (SST)	Speech features	ANN based on autoencoder and data augmentation FS	Accuracy = 90.57%, Sen = 90.57% ASR achieves comparable or better results than manual transcriptions
30	Yamada *et al.* 2021 [54] Japan	Multimodal behavioural data in MCI	45 with MCI (17f, 74.1y), 47 CU (30f, 72.3y)	ASR transcriptions Five speech tasks: countdown subtraction, phonemic and SVF, and CTPD	Several acoustic, prosodic and linguistic features	ML classifiers: SVM, KNN and RF 10-fold CV	AUC = 91.0%, accuracy = 83.0%, Sen = 82.4%, Spe = 83.60%.
31	Wang T *et al.* 2021 [55] China	Speech analysis in MCI	50 with MCI (31f, 63.44y), 60 CU (45f, 64.98y)	Three speech tasks: picture description, SVF and sentence repetition	Multiple lexical, semantic, temporal and acoustic features	ML classifiers: SVM, RF and LR 5-fold CV, FS	Accuracy = 95.0%, Sen = 94.0%
32	Tang *et al.* 2021 [56] USA	Early MCI detection	15 with MCI (8f, 79.3y), 17 CU (14f, 80.0y)	Performing 30 commands of the remote Alex device	Multiple linguistic and acoustic features	ML classifier: LR CV, FS	AUC = 82.7 based on ensemble modelling
33	Kim *et al.* 2021 [57] Korea	Speech markers in MCI	98 with aMCI (53f, 71.0y),104 CU (45f, 70.0y)	SST, picture description and sentence repetition tasks	10 speech metrics, e.g. adding, breathing, continuous, interrupting, laughing, etc.	Traditional statistics	A higher number of pauses and mumbles in MCI
34	Gosztolya *et al.* 2021 [58] Hungary	Acoustic speech markers in MCI	English: 14 with MCI (8f, 72.36),19 CU (14f, 74.47y)	SST, previous day experiences	Seven acoustic: articulation rate, speech rate, utterance duration, pause duration rate, average pause duration, pause frequency	ML classifier: libSVM Nested CV	English: Accuracy = 84.4%, AUC = 93.2%, Sen = 85.7%, Spe = 84.2%
35	Calza *et al.* 2021 [59] Italy	Linguistic markers in MCI	32 with MCI (NR, 64.32y), 48 CU (NR, 61.60y)	Combined ASR and manual transcription Three speech tasks	87 acoustic, rhythmical, morpho-syntactic and lexical	ML classifiers: SVM, RF NLOSOCV FS	An F1 of 75.0% in distinguishing MCI The F1 score is a performance metric that balances precision and recall
36	Nagumo *et al.* 2020 [60] Japan	Automated speech analysis in MCI	468 with MCI (206, 78.5y), 6343 CU (3685, 61.60y)	Temporal characteristics of speech production, planning and processing speed	Temporal (duration of utterance, number and length of pauses) and spectral (F0, F1 and F2) acoustic features	ML classifiers LR 5-fold CV	AUC = 61.0%
37	Chen *et al.* 2020 [30] USA	Automated speech analysis in MCI	28 with MCI (14f, 91.2y), 42 CU (27f, 89.9y)	Combined ASR and manual transcription. Speech fluency task (animals)	Several count-based (total number of unique animal words, switches, etc.) and time-based (switching, etc.) features	ML classifier: SVM FS	AUC = 77.76%, Sen = 76.02, Spe = 67.11
38	Shinkawa *et al.*2019 [61] Japan	Automated speech analysis in MCI	15 with MCI (8f, 74.87y), 19 CU (12f, 71.63y)	Combined ASR and manual transcription CTPD	49 linguistic features categorised into parts of speech, information units, syntactic complexity, sentence similarities and vocabulary richness	ML classifier: SVM LOSO CV	Accuracy = 76.5%, Sen = 73.3%, Spe = 78.0%
39 Fet	Kim *et al.* 2019 [62] Korea	Speech measures for MCI detection	22 with MCI (16f, 70.1y), 21 CU (18f, 71.90y)	Biographical narrative and picture description tasks	Six categories of features: Coherence, cohesion, preposition, grammatical, lexical and fluency	Traditional statistics	Reporting AUC per feature, ranging between 71.8% and 85.9%
40	Fraser et al. 2019 [63] Sweden	Speech analysis in MCI	26 with MCI (14f, 70.6y), 29 CU (21f, 67.8y)	Three language tasks: Picture description, reading silently and reading aloud	Multiple linguistic (26) and acoustic (11) features	ML classifier: SVM, LR	AUC = 71.0%, accuracy = 67.0%, Sen = 55.0%, Spe = 0.79
41	Tóth *et al.* 2018 [64] Hungary	Automatic detection of MCI	48 with MCI (32f, 73.8y), 32 CU (23f, 64.13y)	Both ASR and manual transcriptions SST by answering questions about 2 short films	Acoustic: hesitation ratio, speech tempo, length and number of silent and filled pauses, length of utterance	ML classifier: NB, SVM, RF	AUC = 67.6%, accuracy = 75.0%, Sen = 81.3%, Spe = 66.7%. The accuracy scores for manual and ASR transcriptions were comparable.
42	Nikolai *et al.* 2018 [65] Czech Republic	VF tasks in SCD	61 with SCD (32f, 72.1y), 93 CU (45f, 74.0y)	PVF with letters K and P, and SVF with animal and vegetable categories	Total score, two 30-second intervals, clustering and switching indices	Traditional statistics	SCD generated fewer words in the total score and 30–60-second intervals in the vegetable category, with more switches in the animal category
43	De Looze *et al.* 2018 [66] Ireland	Altered speech chunking in reading aloud in MCI	16 with MCI (NR), 36 CU (NR) >65y	Reading sentences vary in length and syntactic complexity	Temporal acoustic metrics: speech speed (number of speech chunks), silent pauses, fluency, articulation rate	Traditional statistics	AUC = 75.0% Speech chunking in the context of high cognitive-linguistic demand as a marker of MCI
44	Beltrami *et al.* 2018 [25] Italy	Speech analysis with NLP in MCI	16 with MCI (NR, 64.50y), 16 with aMCI (NR, 64.19y), 48 CU (NR, 61.60y)	SST: describing a complex picture, a typical working day and a last dream	Multiple lexical, acoustic and syntactic features	Traditional statistics	Acoustic features can distinguish between MCI and CU, and lexical, rhythmic and syntactic features may also be relevant
45	Mueller *et al.* 2018 [67] USA	Connected language analysis in MCI	200 with MCI (141f, 61.1y), 64 CU (36f, 64.2y)	CTPD	Several semantic, syntax, lexical and fluency features	Traditional statistics	Significant differences in baseline fluency and naming tests, and a faster decline in fluency and semantic content features
46	Asgari *et al.* 2017 [68] USA	Detecting MCI with SSTs	14 with MCI (12f, 61.1y), 27 CU (17f, 64.2y)	Narrative language samples from interviews	Multiple linguistic features from LIWC	ML classifier: SVM, RF 5-fold CV	AUC = 72.5%, Accuracy = 72.4%, Sen = 72.2%, Spe = 72.4%
47	Mueller *et al.* 2016 [69] USA	Connected speech in MCI	39 with aMCI (22f, 63.1y), 39 CU (22f, 63.1y)	CTPD	Semantic and lexical richness, syntactic complexity and fluency	Traditional statistics	Lower scores in the number of semantic units and unique words
48	Aramaki *et al.* 2016 [26] Japan	Vocabulary size in MCI	8 with MCI (3f, 80.25y), 14 CU (7f, 77.21y)	Writing and talking about one of the happiest past events.	Type-token ratio, dependency distance, Yngve score, vocabulary size and level	NLP-based analysis	Larger vocabulary size in MCI, potentially due to engaging in behaviour to slow down cognitive decline. No difference in writing abilities.
49	König *et al.* 2015 [70] France	Automatic speech analysis in MCI	23 with MCI (12f, 73.0y), 15 CU (9f, 72.0y)	Four spoken tasks: countdown, sentence repetition, image description, VF	Several features under voice, silence, periodic and aperiodic segment length categories	ML classifier: SVM Random sub-sampling-based CV, FS	Accuracy = 79.0%
50	Kato *et al.* 2015 [71] Japan	Speech analysis in MCI	75 with MCI (52f, 72.1y), 81 CU (66f, 69.6y)	Questions about birthplace, school name and three digits backward	128 different acoustic features, both segmental and suprasegmental	ML classifiers: LR LOSO CV, FS	AUC = 77.0%, accuracy = 76.5%, Sen = 79.01%, Spe = 75.31%
51	Drummond *et al.* 2015 [72] Brazil	Narrative discourse deficits in MCI	22 with aMCI (11f, 72.1y), 41 CU (26f, 69.6y)	Narrative discourse, confrontation naming and VF tasks.	Narrative type, total number of words, discourse type, etc.	Traditional statistics	Significant differences in repeated words, micro-propositions, IDE, narrative completeness, naming, SVF and PVF
52	Satt *et al.* 2014 [73] France	Automatic speech analysis in MCI	23 with MCI (NR), 15 CU (NR) >65y	Countdown, picture description, sentence repetition, VF tasks	Several features under voice, silence, periodic and aperiodic segment length categories	ML classifier: SVM, NB CV, FS	Accuracy = 76.0%
53	Wutzler *et al.* 2013 [74] Germany	Anticipatory proportion in MCI	20 with MCI (12f, 78.6y), 20 CU (16f, 75.5y)	Tongue twister, Regensburg Word Fluency and object naming tests	AP calculated from the tongue twister task	Traditional statistics	Impaired anticipatory proportion (a marker for error numbers in speech corpora) in MCI.
54	Roark *et al.* 2011 [75] USA	Speech analysis in MCI	37 with MCI (NR, 89.3), 37 CU (NR, 88.8y)	Combined ASR and manual transcription A spoken narrative recall task	Pause frequency and duration, and many linguistic complexity measures	NLP. ML classifier: SVM LOSO CV	AUC = 86.1%

ADNI4, Alzheimer’s disease neuroimaging initiative 4; aMCI, amnestic MCI; ANN, artificial neural network; AUC-ROC, area under the curve -receiver operating characteristics; ASRT, automated story recall task; BCCD, the brief test of cognitive-communication disorders; BNT, Boston naming test; CAT, CatBoost classifier; CoreLex, core lexicon; COWAT, controlled oral word association test; CTPD, Cookie Theft picture description; ; DTW, dynamic time wrapping; f, female; FPR, filled pauses ratio; FS, feature selection; G-match, generalised matching score; GRBF, Gaussian radial basis function; IDE, index of discourse effectiveness, GBM, gradient boosted machines; GNB, Gaussian Naive Bayesian; GI, global index, KNN, k-nearest neighbour; LightGBM, light gradient boosting machine; LIWC, linguistic inquiry and word count; LOSO, leave-one-subject-out; LR, logistic regression; O/CCWR, open/closed class word ratio; MFCC, mel-frequency cepstral coefficients; MLP, multilayer perception; NB, Naïve Bayes classifier; NLF, noun lexical frequency; NLOSOCV, nested leave one-speaker-out cross validation; PID, propositional idea density; RACLT, Rey auditory verbal learning test; RF, random forest; PVF, phonemic verbal fluency; Rep, repletion ratio; RevR: revision ratio; Sn, sensitivity; Sp, specificity; SST, spontaneous speech task; SVF, semantic verbal fluency; VVAS, voice assistant system; VF, verbal fluency; WRR, word replacement ratio.

Table 2 offers a more detailed characterisation of the articles, demonstrating the potential of speech analyses to differentiate MCI from CU. The studies varied considerably in methodology, encompassing both the types of tasks administered and the breadth of speech features investigated. While some studies reported results separately for linguistic or acoustic features, there were inconsistencies in categorising features across the articles, and in several cases, only combined results were presented. These limitations prevented us from conducting subgroup MAs by feature category or task type.

Findings from four articles [5–7, 65] that included an SCD group indicated that speech feature analysis has the potential for detecting subtle cognitive changes in individuals with SCD. However, only one of these studies reported AUC (90.0%) [6].

### Methodological quality of evidence

Based on the JBI critical appraisal checklist, 52 of the 54 included articles were evaluated as high quality, while 2 were rated as moderate quality. Thus, most of the studies were determined to have high methodological quality (see Supplementary Appendix S3).

#### Meta-analysis

**Figure 2:**
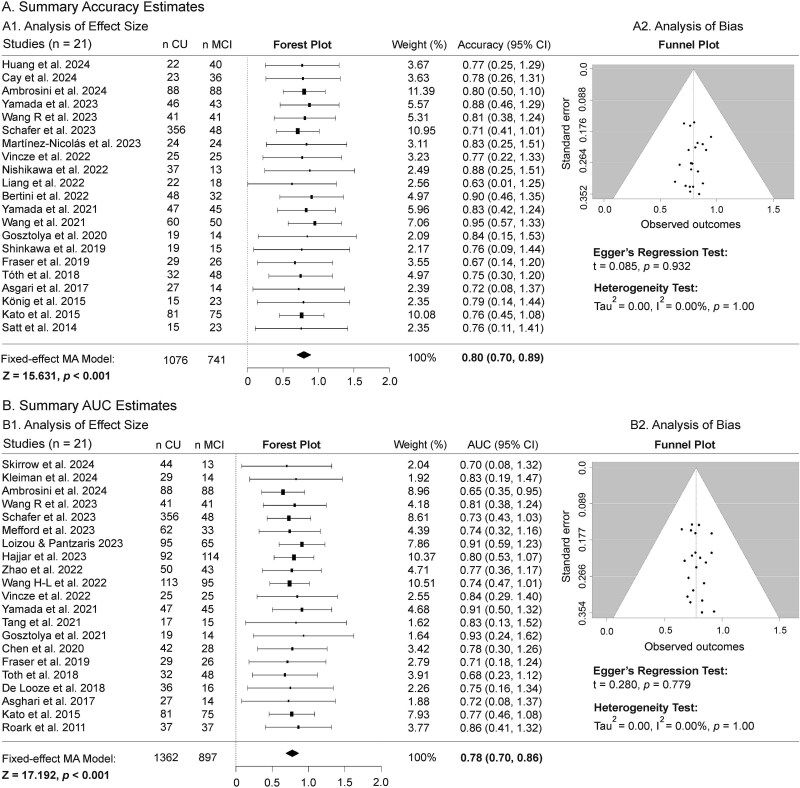
MA of accuracy (A) and AUC (B) performance metrics for differentiating individuals with MCI from CU peers. Panels A1 and B1 present the effect size analyses. The forest plots display the effect size of accuracy or AUC for each study (black boxes with 95% CIs) and the pooled estimate (black diamond). A fixed-effects MA model shows an overall accuracy of 80.0% (95% CI: 70.0%–89.0%) and an overall AUC of 78.0% (95% CI: 0.70%–0.86%), demonstrating that speech analysis can effectively distinguish MCI from CU (*P* < .001). Panels A2 and B2 present the bias analyses. The Funnel plots show that the studies’ SEs are symmetrically distributed around the midline. The *I*^2^ statistic indicates no heterogeneity among studies, and Egger’s regression test indicates no publication bias or funnel plot asymmetry.

**Figure 3:**
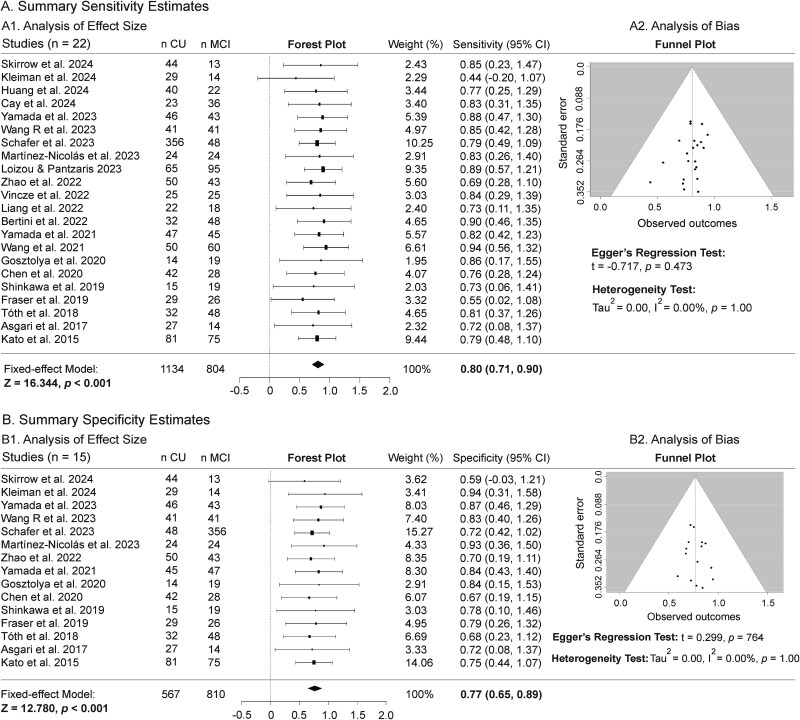
MA of sensitivity (A) and specificity (B) performance metrics for differentiating individuals with MCI from CU peers. Panels A1 and B1 present the effect size analyses. The forest plots display the effect size of sensitivity or specificity for each study (black boxes with 95% CIs) and the pooled estimate (black diamond). A fixed-effects MA model shows an overall sensitivity of 80.0% (95% CI: 71.0%–90.0%) and an overall specificity of 77.0% (95% CI: 0.65%–0.89%), demonstrating that speech analysis can distinguish MCI from CU (*P* < .001) with comparable sensitivity and specificity. Panels A2 and B2 present the bias analyses. The Funnel plots show that the studies’ SEs are predominantly distributed around the midline. The I^2^ statistic indicates no heterogeneity among studies, and Egger’s regression test indicates no publication bias or funnel plot asymmetry.

Of the 53 articles on individuals with MCI, classification performance for accuracy, AUC, sensitivity and specificity was reported in 21, 23, 22 and 15 articles, respectively. To account for the potential impact of imbalanced data sizes, the two studies with the largest sample sizes [46, 60] were excluded from the MA of AUC. In Figures 2 and 3, the forest plot with the diamond marker indicates the pooled estimate of values, and its horizontal tips represent the 95% CI. As shown in the funnel plots, the SEs of the four classification metrics are symmetrically distributed around the middle line of the white inverted funnel, indicating no publication bias among the included studies.

##### Accuracy

In Figure 2A, the pooled effect size analysis indicates 80.0% (95% CI 70.0%–89.0%, *Z* = 15.631, *P* < .001) accuracy in distinguishing MCI from CU. Egger’s regression test demonstrates no publication bias (*t* = 0.085, *P* = .932), and the *I*^2^ test shows no heterogeneity across the included studies (*I*^2^ = 0.00%, *P* = 1.00).

##### Area under the curve

The pooled effect size of 78.0% (95% CI 70.0%–86.0%, *Z* = 17.192, *P* < .001) revealed that AUC significantly distinguishes between MCI and CU (Figure 2B). Egger’s regression test indicates no publication bias (*t* = 0.280, *P* = .779), and the *I*^2^ test demonstrates no heterogeneity across the included studies (*I*^2^ = 0.00%, *P* = 1.00).

##### Sensitivity and specificity

Figure 3A and B indicates a pooled estimate of 80.0% sensitivity (95% CI 71.0%–90.0%, *Z* = 16.334, *P* < .001) and 77.0% sensitivity (95% CI 65.0%–89.0%, *Z* = 12.780, *P* < .001) in distinguishing MCI (*n* = 804) from CU (*n* = 1134). Analyses exhibit no publication bias (*P* ≥ .299) and no heterogeneity across the included studies (*I*^2^ = 0.00%, *P* = 1.00).

## Discussion

Our results demonstrate moderate classification performance for accuracy, AUC, sensitivity and specificity in distinguishing MCI from CU using speech biomarkers, with minimal evidence of publication bias and heterogeneity across studies. Among the included articles, four also examined individuals with SCD, and their findings suggest that speech-based biomarkers may serve as early indicators of cognitive decline. Overall, the methodological quality of the included studies was high. These findings are discussed in detail below.

### Speech-based biomarkers for mild cognitive impairment

Our MA of classification performance using accuracy and AUC provides supporting evidence for the potential of speech-based biomarkers to distinguish MCI from CU. The pooled accuracy of 80.0% (95% CI: 70.0%–89.0%) indicates that, on average, speech-based analyses correctly classify four out of five individuals as either MCI or CU. The pooled AUC of 76.0% (95% CI: 70.0%–86.0%) reflects moderate discriminatory power, consistent with established thresholds for classification performance [35]. Furthermore, the pooled sensitivity of 80.0% suggests that speech biomarkers correctly identify 8 in 10 individuals with MCI, while the pooled specificity of 77.0% indicates that approximately three-quarters of CU individuals are accurately recognised. Taken together, these findings suggest that speech-based biomarkers provide moderate yet promising diagnostic value for detecting MCI, with balanced sensitivity and specificity supporting their potential role in the early identification of cognitive decline.

### Advancement in speech-based analyses: natural language processing, automatic speech recognition and machine learning models

Advances in AI technologies and the integration of ASR and NLP to automated analyses have significantly evolved the research on developing speech-based biomarkers for disease diagnosis, including MCI and AD [46]. Early research in this area often relied on manual transcription and linguistic analysis, which were time-consuming, costly and required trained research staff, limiting their feasibility for large-scale screening. Current research leverages ASR to address these challenges, enabling accurate, automated transcription of spoken language and reducing the need for human involvement [46, 64]. With the integration of automated transcription, researchers can now efficiently extract acoustic features such as speech rate, articulation patterns, pauses, tempo, voice periodicity, hesitation frequency and shimmer, which are increasingly recognised as potential early indicators of cognitive decline [14, 20, 48, 58, 64]. Applying NLP techniques to automated transcriptions furthers this capability, enabling deeper analysis of lexical, syntactic and semantic features that may precede clinically apparent cognitive decline. Advancements in data analysis and the integration of ASR and NLP with ML models have also accelerated this process, allowing for the classification of speech-derived features and the prediction of cognitive status with increasing precision [18, 44, 46]. In addition, ongoing monitoring of speech patterns could be implemented in tools such as smartphone applications to detect early cognitive changes, contributing to the clinical relevance of speech biomarkers [76, 77]. Although these technological advancements promise cost-effective diagnostic markers, their application as universal screening tools in diverse, large-scale populations remains uncertain due to several concerns. First, models trained primarily on English-speaking populations tend to exhibit reduced sensitivity when applied to speakers from diverse linguistic and cultural backgrounds [78]. Second, variations in dialect, education level and socioeconomic status can influence language patterns [79]. Third, challenges remain regarding reaching consensus on ML feature extraction methods [80]. Fourth, findings from specialised clinical settings, such as memory clinics, may not necessarily apply to community-dwelling populations and may lead to misleading conclusions if not carefully contextualised. Therefore, further research is necessary to establish consistent research protocols and assess the reliability and generalisability of the current models.

### Overfitting in machine learning-based speech analysis

Overfitting is a significant challenge in ML-based analyses. It refers to a situation in which a model learns patterns specific to the training data, including noise and outliers, rather than being focused on generalisable features [81]. This phenomenon results in high performance on the training dataset but poor generalisation to unseen data. Excessive model complexity, small or unrepresentative datasets and inadequate regularisation are known factors that contribute to overfitting. The CV technique helps address this by partitioning the data into multiple subsets to validate model performance on unseen data, thereby improving reliability and reducing bias. FS minimises overfitting by retaining only the most informative variables, thereby lowering model complexity, reducing noise and enhancing interpretability. Ensemble methods, including bagging and boosting, mitigate overfitting by combining multiple models, which decreases variance, increases robustness and improves generalisation [81, 82].

A low sample size is a known cause of overfitting. In this review, 25 out of 54 studies had fewer than 30 participants per study group. To address the small-study effect, reviewed studies utilised techniques such as CV, FS, ensemble models or their combination. Although applying these mitigation techniques enhances the overall credibility of our MA results, addressing issues such as sample size, model complexity and validation techniques remain critical for improving the robustness and reliability of speech-based biomarkers in the clinical setting.

### Speech-based biomarkers for subjective cognitive decline

Current longitudinal research indicates that individuals with SCD face a 40%–62% increased risk of progressing to MCI or AD within 3 years [8, 83, 84]. In this review, we identified four studies that used speech-based biomarkers to differentiate SCD from CU, with their results suggesting that subtle linguistic and acoustic alterations in SCD may serve as early indicators of cognitive decline [5–7, 65]. For example, in the study by Nikolai *et al.* (2018) using verbal fluency tasks, individuals with SCD produced fewer words in the vegetable category (e.g. season, botanical family, manner of eating) and showed increased switching in the animal category (e.g. habitat, zoological family, family relation) compared to the CU group. This finding was interpreted as early deficits in both semantic retrieval and executive function [65], reflecting cognitive challenges in SCD. Similarly, König *et al.*’s (2024) study showed the feasibility of an automated word count feature for detecting cognitive decline. However, it did not report pairwise comparisons, limiting the ability to determine whether speech analysis can distinguish SCD from both CU and MCI [5].

Two of the included studies used acoustic and prosodic markers for SCD detection. He *et al.* (2023) reported an AUC of 90% in distinguishing SCD from CU. The ML analysis demonstrated that even minor speech variations can provide strong classification performance [6]. Tröger *et al.* (2022) also found significant differences among MCI, SCD and CU groups using ASR-based fluency and recall tasks. However, due to the lack of pairwise comparisons, the model’s ability to distinguish between subgroups was uncertain [7]. While these findings suggest that speech features can detect subtle cognitive changes in SCD, methodological diversity observed limits the ability to draw firm conclusions, indicating the need for further research.

### Comparison with neuroimaging evidence

Neuroimaging techniques have been extensively studied for their ability to distinguish MCI or AD from CU. In an SR and MA by Ruan and Sun (2023), PET amyloid-β findings showed high sensitivity (0.91) and specificity (0.81) in differentiating AD from CU [85]. Two fluorodeoxyglucose PET studies in patients with pathologically confirmed dementia reported diagnostic accuracies of 82% [86] and 89.6% [87] compared to CU. In ML-based analyses of resting-state and task-based EEG data to distinguish MCI from CU, an SVM classifier achieved an AUC of 87.0% and an accuracy of 76.0%, while follow-up assessments reported an AUC of 75.0% and an accuracy of 70.0% [88]. Overall, MAs and individual studies consistently show a slightly higher classification accuracy of neuroimaging methods in differentiating AD from CU than in distinguishing MCI from CU [85]. Our MA findings on speech-based biomarkers align with existing neuroimaging evidence in differentiating MCI from CU.

### Limitations and directions for future research

Despite our findings supporting the potential of speech-based biomarkers for detecting MCI and SCD, several limitations must be acknowledged. A key challenge lies in the diversity of study methodologies, including variations in speech tasks, speech parameters, feature extraction techniques and ML models, all of which significantly influence outcomes. Variability in data collection and analysis methods also complicates the development of models that can be universally applied. Furthermore, several included studies had relatively small sample sizes, increasing the risk of overfitting. These limitations highlight that speech biomarkers for detecting cognitive decline remain an emerging field and require further research to refine methods and improve generalisability.

In addition, the diagnostic utility of biomarkers should be tested in large and demographically diverse populations to enhance generalisability. Intra-individual variability is also understudied; fluctuations in fatigue, mood, attention or other transient states can affect model performance. To address this, future research should adopt longitudinal designs and incorporate repeated assessments across multiple time points to evaluate the temporal reliability of speech metrics. Integrating wearable speech monitors with remote assessment platforms also hold significant potential to improve the reach and accessibility of speech-based biomarkers.

Another consideration is the interpretation of speech-based features as biomarkers. Some researchers argue that these features may serve better as indicators of elevated risk rather than as direct representations of underlying disease mechanisms, reflecting the distinction between correlational markers and mechanistic biomarkers [89–91]. Whereas structural imaging or CSF markers may directly reflect neuropathology, speech-based features often capture behavioural changes that can be more variable and less specific. However, this challenge is not unique to speech analysis; it extends to many emerging markers, such as gait variability, grip strength and reaction time, which also do not directly index disease pathology. Importantly, the clinical relevance of such biomarkers is supported by their non-invasiveness, cost-efficiency and sensitivity to subtle changes that precede clinical diagnosis. Future research adopting a broader multimodal framework may help clarify the clinical utility of speech features for the early detection of cognitive decline and for guiding interventions.

## Conclusions

By synthesising findings from 54 studies, with 35 contributing to MAs, our results demonstrate that speech analysis can differentiate MCI from CU. Our findings demonstrate that speech-based biomarkers achieve moderate but promising diagnostic performance in distinguishing MCI from CU, with balanced accuracy, sensitivity, specificity and AUC values. These results highlight the potential of speech analysis as a non-invasive and accessible tool for the early identification of cognitive decline. Important methodological advancements, such as integrating ASR, NLP and ML techniques, have significantly enhanced the speech features ‘precision and utility. Despite these advancements, challenges remain, including variations in research methodologies, the risk of overfitting in ML models due to small sample sizes and potential biases resulting from training datasets. While CV techniques are widely used to mitigate overfitting, further research is needed to refine speech analysis protocols and use larger, more diverse datasets to improve generalisability.

## Supplementary Material

aa-25-1358-File005_afaf316
